# A comparative cross-cultural study of the prevalence of late life depression in low and middle income countries

**DOI:** 10.1016/j.jad.2015.09.004

**Published:** 2016-01-15

**Authors:** M. Guerra, A.M. Prina, C.P. Ferri, D. Acosta, S. Gallardo, Y. Huang, K.S. Jacob, I.Z. Jimenez-Velazquez, J.J. Llibre Rodriguez, Z. Liu, A. Salas, A.L. Sosa, J.D. Williams, R. Uwakwe, M. Prince

**Affiliations:** a Institute of Memory, Depression and Disease Risk, Avda Constructores 1230, Lima 12, Peru; b Centre for Global Mental Health, Health Service and Population Research Department, Institute of Psychiatry, Psychology and Neuroscience, King׳s College London, London, UK; c Peruvian University, Cayetano, Heredia, Lima, Peru; d Federal University of Sao Paulo, UNIFESP, Sao Paulo, Brasil; e National University Pedro Henriquez Urena; f Peking University China; g Christian Medical College, Vellore, India; h School of Medicine, University of Puerto Rico, San Juan, Puerto Rico; i Medical University of Havana; j Central University of Venezuela, Caracas, Venezuela; k National Autonomous University of Mexico; l Nnamdi Azikiwe Uniiversity; m Department of Community Health, Voluntary Health Services, Chennai, India

**Keywords:** Depression, Prevalence, ICD-10, EURO-D, Older-age

## Abstract

**Background:**

Current estimates of the prevalence of depression in later life mostly arise from studies carried out in Europe, North America and Asia. In this study we aimed to measure the prevalence of depression using a standardised method in a number of low and middle income countries (LMIC).

**Methods:**

A one-phase cross-sectional survey involving over 17,000 participants aged 65 years and over living in urban and rural catchment areas in 13 sites from 9 countries (Cuba, Dominican Republic, Puerto Rico, Mexico, Venezuela, Peru, China, India and Nigeria). Depression was assessed and compared using ICD-10 and EURO-D criteria.

**Results:**

Depression prevalence varied across sites according to diagnostic criteria. The lowest prevalence was observed for ICD-10 depressive episode (0.3 to 13.8%). When using the EURO-D depression scale, the prevalence was higher and ranged from 1.0% to 38.6%. The crude prevalence was particularly high in the Dominican Republic and in rural India. ICD-10 depression was also associated with increased age and being female.

**Limitations:**

Generalisability of findings outside of catchment areas is difficult to assess.

**Conclusions:**

Late life depression is burdensome, and common in LMIC. However its prevalence varies from culture to culture; its diagnosis poses a significant challenge and requires proper recognition of its expression.

## Highlights

• Late life depression prevalence was evaluate in 17,000 older adults from LMIC.
• Depression prevalence varied across sites according to diagnostic criteria.
• Prospective longitudinal studies are needed in order to clarify aetiological factors.
• Expression of depression in different cultures needs more work and further analysis.
• Prioritisation of recognition and treatment of depression in older adults should be on the agenda of policy-makers across the world.

## 1 Introduction

Depression, a prevalent and extremely disabling psychiatric condition in later life (Beekman et al., 1999, Blazer, 2003), has not been studied sufficiently in low and middle income countries (LMIC) where a demographic transition, with an increasing number of older people is rapidly occurring (Christensen et al., 2009).

In high-income countries, the prevalence of late-life depression has been extensively studied (Beekman et al., 1999, Djernes, 2006) with a considerable variation reported across studies, with the operational criteria being a main influence.

To our knowledge at least 21 studies have been conducted from 1990 until 2011 in LMIC using different criteria. Most of the studies were carried out in China (Chen et al., 1999, Chen et al., 2004, Chen et al., 2005, Meng and Tang, 2000, Pan et al., 2008, Wu and Zhang, 1989), or Latin America {Zunzunegui et al., 2009 #1409; Alvarado et al., 2007 #118; Costa, 2007 #130; Blay and Marinho, 2007 #131;García-Peña et al., 2008 #1343;Carvalhais et al., 2008 #1223; Tintle et al., 2011 #1299;Guerra et al., 2009 #120; Barcelos-Ferreira, 2010 #1099}. The majority of Latin American studies had a small sample size and used depression symptom scales and reported a relatively higher prevalence of depression, compared to those studies from Mainland China.

One of the biggest multicentre studies (SABE) was conducted in six Latin American capital cities using the Geriatric Depression Scale, and reported a depression prevalence ranging from 16.5% to 30.1% in women and from 11.8% to 19.6% in men (Alvarado et al., 2007); results that are broadly consistent with estimates from two cross-national comparisons of late-life depression in Europe: SHARE (Castro-Costa et al., 2007) and EURODEP. In the 10/66 population based study conducted in Peru, Mexico and Venezuela, the prevalence varied depending on the diagnoses criteria used being higher for GMS–AGECAT (between 30.0% and 35.9%) and EURO–D depression scale (cutpoint3/4) (between 26.1% and 31.2%).

We now extend the evidence of the prevalence of late-life depression to include a wider range of settings, in Latin America, Nigeria and Asia..

## 2 Methods

### 2.1 Setting, design and procedures

The 10/66 Dementia Research Group population-based studies were all conducted according to the same standardised protocol. The full 10/66 study protocol has been published elsewhere (Prince et al., 2007). A one-phase cross-sectional population-based survey has been conducted of all those over 65 years old from defined catchments areas. Surveys were carried out in thirteen sites in nine countries (Cuba, Dominican Republic, Puerto Rico, Peru, Mexico, Venezuela, China, India and, Nigeria). Surveys in Peru, Mexico, China and India included both urban and rural catchment areas, the Nigerian catchment area was predominately rural, while in the other countries participants were recruited only from urban catchment areas.

All assessments were carefully translated and adapted into the relevant local languages. Acceptability and conceptual equivalence were assessed and reviewed by local informants. Interviews were carried out in participants’ own homes and lasted on average two to three hours. Interviewers were fully trained on the 10/66 protocol by the local principal investigator (PI) and the local study coordinator (SC). The study protocol and the consent procedures, including the witnessed consent procedure, were approved by the King's College London research ethics committee and in all local countries.

Funding for each group of countries was obtained at different times, therefore these baseline surveys were conducted over a six year period (2003–2009).

## 3 Measurements

### 3.1 Depression

Depression was determined according to EURO-D and ICD-10 criteria, all generated from the same semi-structured clinical interview, the Geriatric Mental State (GMS), which is supported by the computerised diagnostic algorithm AGECAT (Automated Geriatric Examination for Computer Assisted Taxonomy) (Copeland et al., 1976). For all criteria, period prevalence was determined with respect to the last one month.

#### 3.1.1 Depression of clinical significance

The EURO-D (Prince et al., 1999) is a symptom scale that covers 12 symptom domains: depressed mood, pessimism, suicidality, guilt, sleep, interest, irritability, appetite, fatigue, concentration, enjoyment and tearfulness. Each item is scored 0 (symptom not present) or 1 (symptom present), and item scores are summed to produce a scale with a minimum score of zero and a maximum of 12. The EURO-D scale had moderately high internal consistency in the EURODEP study (Prince et al., 1999), and was reported to have good construct validity in the our 10/66 sample (Brailean et al., 2015). For this study, we determined the optimal- cutpoint in each site (as either 4 or 5), as described in the EURO-D validation paper that we have recently published (Guerra et al., 2015). In summary, the optimal cutpoint, its sensitivity and specificity were respectively: Cuba (cutpoint: 5, sensitivity at cutpoint: 97.2%, specificity 87.7%), Dominican Republic (5, 93.5%, 84%), Puerto Rico (5, 97.9%, 91.6%), urban China (6. 100%, 97.8%), rural China (5, 85.7%, 99.6%), urban India (5, 97.4%, 74.1%), India rural (4, 91.3%, 69.5%) and Nigeria (5, 100% 79.3%)

#### 3.1.2 Diagnostic criteria for depression

ICD-10 diagnoses were derived using a computerised algorithm applied to the GMS. For ICD-10, F32 Depressive episode, specified as mild, moderate or severe was used.

### 3.2 Socio-demographic status and other health-conditions

Age was established during the interview from the participant using official ID documentation, informant report, and, in the case of discrepancy an event calendar was used. We also obtained information on: gender and marital status (single, married/ cohabiting, widowed, divorced/separated); education (none, did not complete primary, completed primary, secondary, tertiary); social support (living alone versus living with others; frequency of contact with relatives and friends); occupational attainment (professional, clerical or trade, skilled or semi-skilled manual worker); amount and sources of income; number of assets, and food insecurity.

Other health conditions were self-reported (e.g. angina, stroke, COPD, etc.), diagnosed (e.g. dementia using the 10/66 dementia algorithm (Prince et al., 2003), or determined according to specific criteria (e.g. hypertension).

## 4 Statistical analysis

We used the 10/66 data archive (release 3.0) and STATA (version 11 or 13) for all analyses. The prevalence of depression, accompanied by robust 95% confidence intervals (CIs), was estimated in Cuba, Dominican Republic, China, India, Nigeria and Puerto Rico.

Direct standardisation estimates (for age, sex and education), using the whole sample as the standard population, were also reported for all the sites, including the sites where we previously published non-standardised estimates (Peru, Mexico and Venezuela){Guerra et al., 2009 #120}.

In each setting, we report the prevalence of depression with 95% confidence intervals, by age and sex, for both ICD-10 depressive episode and EURO-D depression (4/5 cut-point).

Forest plots from a random effect meta-analysis were generated using the *metaprop* command in STATA for both ICD-10 and EURO-D estimates, and reported with their pooled estimates.

In order to explore the risk of age and gender on prevalent ICD-10 depression, we used Poisson regressions to calculate mutually adjusted prevalence ratios (PRs). We then used a fixed-effect meta-analysis to pool the PRs across sites, also reporting an I^2^ Higgins score to highlight the heterogeneity across sites.

The prevalence of ‘sub-syndromal depression’ was also reported. This was defined as those not meeting criteria for ICD-10 depressive episode, but scoring above the optimal cut-point on the EURO-D scale.

## 5 Results

### 5.1 General characteristics

Overall, 17,852 interviews were completed. Response proportions ranged from 72% (urban India) to 98% (rural India). General characteristics of the respondents in each country are summarised in Table 1. Women predominate over men in all sites, with nearly two- thirds of participants being women in Latin American sites, and just over a half in China, India and Nigeria. Higher levels of education were registered in Latin America and in urban areas in comparison to rural areas. Participants in rural locations also reported fewer household assets, more food insecurity, and lower personal income, compared to those living in urban locations. Between 1.2% (rural China) and 34.9% (urban Peru) reported a past history of depression.

### 5.2 Prevalence of depression

The largest source of variation in the prevalence of depression was the criterion used for assessment. The prevalence of ICD-10 depressive episode varied between 0.3% and 13.8% by location **(**Table 2**)**, whereas the prevalence of EURO-D depression ranged between 1.0% and 38.6% **(**Table 3). However, for each of these criteria, there was also substantial heterogeneity in prevalence among sites (supplementary fig. 1). The meta-analysed pooled estimate for ICD-10 depression was 4.7 (95% CI: 3.1-6.3) and for EURO-D depression 18.2 (96% CI: 12.3-24.0).

**Fig. 1:**
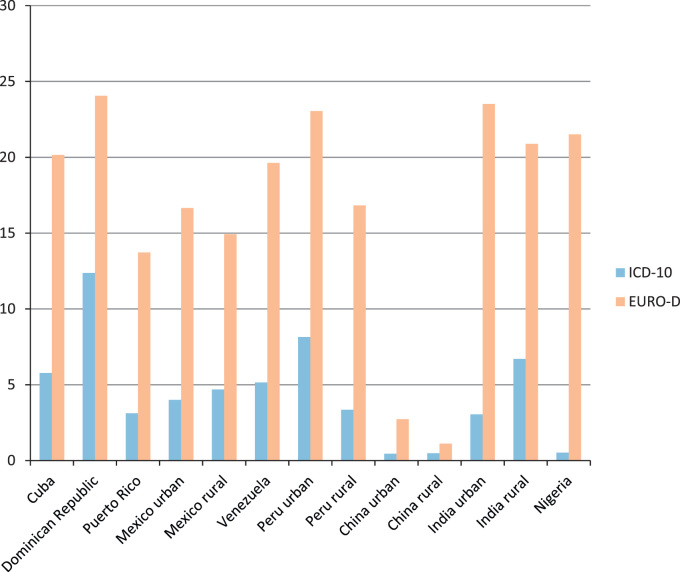
Prevalence of depression (%) using different operational criteria, standardised by age, gender and education.

Direct standardisation had some effect on the estimates, as shown in Fig. 1 which reports the prevalence for both criteria using direct standardisation for age, gender and education. The prevalence in Dominican Republic, with all diagnostic criteria, was high with respect to that observed in other Latin American sites. The prevalence was exceptionally low in urban and rural China with all criteria.

In all sites with exception of rural Peru, rural China and both Indian sites, the prevalence of depression was higher in women than among men. In Latin America, the prevalence of ICD-10 depression increased with age in men, but not in women, whereas an increasing trend in EURO-D prevalence was seen across both genders and sites.

When we adjusted for both age and gender and pooled our estimates across sites, we found that men, and younger individuals had lower PRs of ICD-10 depression (pooled estimates: 0.62, 95% CI: 0.53–0.71, *I*^2^=0.0% and 1.07, 95% CI=1.02–1.12, *I*^2^=45.2% respectively).

Given the higher prevalence of EURO-D depression compared with ICD-10 depressive episode we explored the the concept of sub-syndromal depression (EURO –D depression not confirmed as a depressive episode by the ICD-10).

The prevalence of sub-syndromal depression varied across sites with urban China having the lowest (0.4%) and rural India the highest (25.3%) (Table 4).

### 5.3 Depression clinical aspects

Overall, 35.3% of ICD-10 depression cases were mild, 51.9% were moderate, and 12.7% severe. The proportion of current ICD-10 depressive episode cases with past history of depression varied between 25.6% and 71.8%, with rural India constituting a low outlier with only 2/126 cases (1.6%) reporting a past history of depression. In general a past history was more frequently reported in urban than rural sites. In Latin American sites, where a past history of depression was relatively frequently reported, around 20% to 60% of these individuals reported having previously been treated by a doctor, with higher proportions in Cuba, Puerto Rico, and Venezuela than in Dominican Republic, Mexico and Peru. The median age for first onset of depression exceeded 60 years for most sites.

## 6 Discussion

In this study, we reported a wide variation of estimates according to the depression criterion that we used. Across all sites, the prevalence of ICD-10 depressive episode was higher than EURO-D depression (a score of 5 or higher on the EURO-D scale). However, for each of these criteria, there was also substantial variation in prevalence among sites. Therefore it is important to compare results between studies, where possible, based on the use of the same or similar criteria. On this basis, our results suggested a higher prevalence of late-life depression, in at least some sites in Latin America, and in urban India, than is typically recorded in studies in high income countries. Conversely, the prevalence in China was very low.

### 6.1 Strengths and weaknesses of the study

To our knowledge this is the first large-scale community-based depression-prevalence study conducted in LMIC that, with the same methodology, has evaluated a large number of older persons, in nine LMIC located in three continents, using rigorous research diagnostic criteria such as the ICD-10 and the EURO-D. Unlike HIC, an important advantage in our study is the relatively high response rate, at least 80% in all sites, and exceeding 90% in several sites. Rather than a comprehensive clinical diagnostic interview depression was determined according to two different criteria (ICD-10; EURO-D). While the findings of this study may be to some extent generalisable to other similar urban or rural sites, they may not be generalised to the whole city, or country where the study was conducted. Comparison of findings with studies that systematically sampled whole cities, or conducted national surveys may be particularly difficult.

### 6.2 Depression prevalence

Other than the relatively high prevalence of ICD-10 depressive episode in Dominican Republic and rural India, and the low estimates of China and Nigeria, our findings are broadly consistent with those reported in high income countries. A review from Djernes and colleagues (Djernes, 2006) reported an ICD-10 prevalence of 3.3% in Australia and 7.7% in Denmark; More recently, a Brazilian community-based survey of older adults (Costa et al., 2007) reported an unusually high prevalence of ICD-10 depressive episode (19.2%). However, it is difficult to compare our findings with this study, since their sample size was small (n=413), people with dementia were excluded, the age range was 75 years and older, and a two phase design (Symptom scale & semi-structured SCAN interview) was used. The prevalence of EURO-D depression was generally six times higher than that of ICD-10 depressive episode. These ratios are consistent with earlier reviews and studies regarding the ratio of depression identified with such screening scales, as compared to clinical diagnoses (Castro-Costa et al., 2007, Prince et al., 2004). A large community study, carried out in ten European countries in persons aged 55 and above, using EURO-D measure reported prevalence rates between 19% and 33% (Castro-Costa et al., 2007). Our results are congruent with these results even though methods differences between studies and there is much more variability in prevalence among sites in our study, mainly arising from the low prevalence in China. Unlike rigid criteria-based instruments (ICD-10 and DSM-IV), identification as a probable case of depression using EURO-D depends only on the overall load of reported symptoms, rather than requiring the presence of particular symptoms and combination of symptoms, and is without regard to their duration, persistence or pervasiveness. As such, it is important to recognise that not all of these individuals would be considered to be ‘cases for treatment’ since current evidence-based recommendations are exclusively for those with moderate to severe case level depression (Patel, 2009). The discrepancy in prevalence between the two approaches is explained by the less than perfect specificity of the EURO-D, which, given the low prevalence of DSM-IV and ICD-10 depression in population settings results in a low positive predictive value. The disparity is striking, particularly in Nigeria, where very few if any clinical diagnoses were recorded, but there was a relatively high prevalence of most depression symptoms, and a high prevalence of EURO-D depression. The generally much higher prevalence of EURO-D depression raises the question “what constitutes a case?”. This issue was discussed in an earlier review of late-life depression in which the disparity between prevalence according to clinical diagnostic criteria (1.8%) and using symptoms scales and other less restrictive criteria (13.5%) was first highlighted (Beekman et al., 1999). Although not all EURO-D cases may be ‘cases for treatment’, reliance upon clinical diagnoses may significantly underestimate the population burden of depression symptoms, much of which may arise from the larger number of individuals with less severe ‘sub-syndromal’ depression.

### 6.3 Variation of prevalence among sites

As can be appreciated from the above, the pattern of variation of prevalence among sites was generally similar for the two diagnostic criteria. Estimates were generally high, and fairly consistent in Latin American sites, lower in urban India than in rural India (where prevalence was similar to that of the highest prevalence Latin American site, the Dominican Republic) and very low in the two Chinese sites. Nigeria was unusual in this respect, with a very low prevalence of ICD-10 depression, but a comparatively high prevalence of EURO-D depression, similar to that in Latin American sites. The low prevalence of depression in China might be partly explained by contextual factors including the influence of culture on ascertainment of depression. In China the once popular and prevalent diagnosis of shenjing shuairuo, a neurasthenia like syndrome comprising weakness, fatigue, concentration problems, headache and other somatic symptoms seems in recent years to have been supplanted as the most common diagnosis in epidemiological surveys and clinical practice by depressive and anxiety disorders (Lee, 1999). This has led some to allege an inappropriate importation of western nosologies that do not match well with Chinese cultural idioms of expression of psychological distress (Lee, 1999). In this context, it is perhaps noteworthy that depression was not a common symptom in either urban or rural Chinese sites, and the sleep disturbance, fatigue and irritability were the three commonest symptoms in the urban site, and tearfulness, lack of concentration and loss of interest in the rural site. More work needs to be done to establish the validity of the GMS interview, across cultures as a tool for generating ICD-10 and EURO-D diagnoses. None of the systematic reviews of studies we used as a guide of the prevalence of depression (Cole MG, 2003, Djernes, 2006) considered the effect of urban or rural residence. In this study there was a trend towards a lower prevalence of late-life depression in rural than urban sites in Latin America, with the opposite trend seen in India. Findings elsewhere in the literature are inconsistent. Some community cross-sectional studies reported a higher prevalence in urban residence (Carpiniello and Rudas N., 1989, Chiu et al., 2005, Gureje et al., 2007), associated with a higher prevalence of chronic medical conditions and functional impairment, and lack of, or poor social support. Others did not find any association (St John et al., 2006).

## 7 Conclusion

Overall our findings are congruent with those previously reported in the literature and given the pattern of findings, we can conclude that late-life depression prevalence varied depending on the criterion used for assessment. Wide variation in prevalence among sites needs to be evaluated. More work needs to be done to understand adequately the expression of depression in different cultures. This must be the focus of further analysis. Prospective longitudinal studies are needed in order to clarify aetiological factors and to disentangle those factors that influence prevalence through increasing the duration of depressive episodes (maintenance of depression) and those that increase the incidence (onset) of depression.

Given the high burden of this condition, prioritisation of recognition and treatment of depression in older adults should be on the agenda of policy-makers across the world. This goes together with the urgent need to strengthen primary care settings, development of locally appropriate support services as an important component of ensuring social protection and finally to develop primary and secondary prevention strategies using evidence from appropriate studies.

## Figures and Tables

**Table 1:** Socio-dtemographic characteristics of the sample.

	**Cuba**	**Dominican Republic**	**Puerto Rico**	**China Urban**	**China Rural**	**India Urban**	**India Rural**	**Nigeria**
	***n*=2944**	***n*=2011**	***n*=1918**	***n*=1160**	***n*=1002**	***n*=1003**	***n*=999**	***n*=914**
**Age (years)**								
** Mean age**	74.8	75.2	76.1	73.9	72.4	71.2	72.5	72.6
** 65–69**	760 (25.8)	533 (26.5)	406 (21.1)	316 (27.2)	383 (38.2)	415 (41.4)	331 (33.1)	386 (42.2)
** 70–74**	789 (26.8)	520 (25.8)	439 (22.8)	362 (31.2)	296 (29.5)	318 (31.7)	350 (35.0)	222 (24.2)
** 75–79**	639 (21.7)	397 (19.7)	456 (23.7)	254 (21.9)	202 (20.1)	144 (14.3)	177 (17.7)	121 (13.2)
** 80+**	749 (25.5)	561 (27.9)	618 (32.1)	228 (19.6)	121 (12.0)	124 (12.3)	141 (14.1)	185 (20.2)
**Missing values**	7	0	2	0	0	2	0	0

**Gender**								
** Female**	1913 (64.9)	1325 (65.9)	1289(67.2)	661 (56.9)	556 (55.4)	571 (57.6)	545 (54.5)	539 (58.9)
**Missing values**	0	2	4	0	0	15	0	0

**Marital status**								
** Never married**	275 (9.3)	139 (6.9)	118 (6.1)	3 (0.2)	22 (2.2)	21 (2.1)	5 (0.5)	41 (4.8)
** Currently married**	1271 (43.2)	586 (29.3)	931 (48.5)	829 (71.4)	585 (58.3)	523 (52.2)	481 (48.1)	581 (68.6)
** Widowed**	928 (31.6)	806 (40.3)	640 (33.3)	326 (28.1)	394 (39.3)	426 (42.5)	497 (49.7)	225 (26.5)
** Separated/divorced**	462 (15.7)	465 (23.3)	228 (11.8)	2 (0.1)	1 (0.1)	32 (3.1)	16 (1.6)	0 (0.0)
**Missing values**	8	15	4	0	0	3	0	67

**Education level**								
** None**	75 (2.5)	392 (19.6)	70 (3.6)	232 (20.0)	579 (57.7)	428 (42.6)	660 (66.0)	543 (59.4)
** Minimal**	655 (22.3)	1022 (51.3)	376 (19.5)	153 (13.1)	114 (11.3)	234 (23.3)	195 (19.5)	135 (14.7)
** Primary**	979 (33.3)	370 (18.5)	395 (20.5)	303 (26.1)	259 (25.8)	212 (21.1)	116 (11.6)	126 (13.7)
** Secondary**	728 (24.8)	135 (6.7)	686 (35.7)	335 (28.8)	45 (4.4)	87 (8.6)	26 (2.6)	20 (2.1)
** Tertiary**	499 (17.0)	73 (3.6)	388 (20.2)	137 (11.8)	5 (0.5)	42 (4.1)	2 (0.2)	18 (1.9)
**Missing values**	8	19	0	0	0	2	0	0

**Living arrangementsAlone**								
** With spouse only**	261 (8.8)	254 (12.6)	472 (23.5)	54 (4.6)	49 (4.8)	44(4.3)	120 (12.0)	
** With adult children**	445 (15.2)	135 (6.7)	666 (33.2)	415 (35.7)	194 (19.3)	108 (10.7)	140 (14.0)	No data
**Any other**	1422 (48.3)	963 (47.8)	548 (27.3)	446 (38.4)	679 (67.7)	719 (71.5)	625 (62.5)
**Missing values**	816 (27.7)	659 (32.7)	323 (16.1)	245 (21.2)	80 (7.9)	134 (13.3)	114 (11.4)
	**7**	0	0	10	11	2	0

**Past depression**	944(32.2)	357(17.8)	428(22.3)	19(1.6)	12(1.2)	24(2.4)	22(2.2)	14(1.7)
**Missing values**	11	3	0	0	0	1	0	0

**Table 2:** Prevalence of depression (%) in each site, according to ICD-10 depressive episode criterion, stratified by age and sex.

**Age groups (years)**	**65–69**	**70–74**	**75–79**	**80+**	**All ages**	**Crude prevalence**
**Cuba**						
Men	1.1 (0.0–2.3)	2.4 (0.6–4.2)	3.0 (0.8–5.3)	4.3 (1.7–6.9)	2.6 (1.6–3.6)	4.9 (4.1–5.7)
Women	6.8 (4.5–9.0)	4.8 (2.9–6.7)	8.1 (5.4–10.7)	5.2 (3.3–7.2)	6.1 (5.0–7.2)	
**Dominican Republic**						
Men	8.5 (4.5–12.5)	6.7 (3.1–10.2)	15.9 (9.6–22.2)	15.4 (9.9–20.9)	11.1 (8.8–13.5)	13.8 (12.3–15.3)
Women	13.4 (10.3–17.6)	13.9 (10.0–17.7)	16.2 (11.8–20.7)	16.8 (13.1–20.6)	15.2 (13.3–17.2)	
**Puerto Rico**						
Men	0.4 (0.0–7.5)	No cases	1.3 (0.5–3.1)	0.9 (0.4–2.2)	1.2 (0.4–2.1)	2.3 (1.7-3.0)
Women	2.6 (0.8–4.5)	1.4 (0.0–2.7)	2.0 (0.4–3.6)	4.2 (2.3–6.2)	2.8 (1.9–3.7)	
**China urban**						
Men	No cases	No cases	No cases	No cases	No cases	0.3 (0.0–0.6)
Women	No cases	0.5 (0.0–1.5)	No cases	1.7 (0.0–4.0)	0.5 (0.0–1.0)	
**China rural**						
Men	0.5 (0.0–1.5)	1.5 (0.0–3.7)	1.3 (0.0–3.9)	No cases	0.9 (0.0–1.8)	0.7 (0.2–1.2)
Women	0.5 (0.0–1.6)	0.6 (0.0–1.8)	No cases	1.3 (0.0–3.9)	0.5 (0.0–1.1)	
**India urban**						
Men	4.0 (1.1–7.0)	2.4 (0.0–5.1)	5.9 (0.1–11.8)	7.7 (0.2–15.2)	4.3 (2.4–6.2)	3.9 (2.7–5.1)
Women	4.6 (1.9–7.3)	4.8 (1.7–7.8)	1.3 (0.0–3.9)	No cases	3.7 (2.1–5.2)	
**India rural**						
Men	12.2 (6.7–17.7)	14.9 (8.2–20.6)	12.5 (5.5–19.5)	12.3 (4.6–20.1)	13.2 (10.1–16.3)	12.6 (10.5–14.7)
Women	10.9 (6.5–15.4)	14.8 (9.–19.8)	10.1 (3.7–16.5)	10.3 (2.9–17.7)	12.1 (9.4–14.8)	
**Nigeria**						
Men	No cases	1.3 (0.0–3.9)	No cases	1.1 (0.0–3.1)	0.5 (0.0–1.3)	0.5 (0.1–1.0)
Women	0.8 (0.0–1.9)	0.7 (0.0–2.0)	No cases	No cases	0.6 (0.0-1.2)	

**Table 3:** Prevalence of depression (%) in each site, according to EURO-D criterion (cutpoint 4/5), stratified by age and sex.

**Age groups (years)**	**65-69**	**70-74**	**75-79**	**80+**	**All ages**	**Crude prevalence**
**Cuba**						
Men	9.5 (6.0–13.0)	8.9 (5.6–12.2)	10.9 (6.8–14.9)	14.2 (9.7–18.7)	9.5 (7.7–11.3)	16.5 (15.1–17.9)
Women	21.8 (18.1–25.4)	18.1 (14.7–21.5)	22.0 (17.9–26.1)	25.4 (21.6–29.1)	20.3 (18.4–22.1)	
**Dominican Republic**						
Men	17.6 (12.1–23.0)	15.4 (10.3–20.5)	25.0 (17.5–32.5)	24.9 (18.3–31.4)	19.6 (16.6–22.5)	26.8 (24.8–28.8)
Women	28.8 (23.9–33.6)	27.2 (22.2–32.1)	31.7 (26.1–37.3)	36.7 (31.9–41.6)	30.6 (28.1–33.2)	
**Puerto Rico**						
Men	14.2 (7.4–30.9)	7.4 (3.1–11.6)	8.9 (4.4–13.5)	13.8 (9.2–18.5)	6.3 (4.4–8.2)	10.6 (9.2–12.0)
Women	7.1 (12.8–21.5)	11.7 (7.9–15.4)	13.3 (9.5–17.2)	23.4 (19.3–27.6)	12.6 (10.8–14.5)	
**China urban**						
Men	2.7 (0.0–5.7)	3.5 (0.9–6.0)	3.4 (0.0–6.8)	11.0 (5.0–16.9)	1.9 (0.7–3.1)	2.5 (1.6–3.4)
Women	4.4 (1.6–7.3)	3.1 (0.4–5.8)	5.1 (1.4–8.8)	12.6 (6.6–18.7)	3.0 (1.7–4.3)	
**China rural**						
Men	3.1 (0.6–5.6)	3.8 (0–7.1)	7.8 (1.7–13.9)	8.7 (0.2–17.2)	1.4 (0.3–2.5)	1.0 (0.3–1.7)
Women	1.6 (0.0–3.4)	3.0 (0.4–5.7)	3.2 (0.0–6.3)	6.7 (0.9–12.4)	0.7 (0.0–1.5)	
**India urban**						
Men	17.3 (11.6–23.0)	18.3 (11.4–25.1)	29.9 (18.6–41.1)	32.7 (19.5–45.9)	20.9 (17.0–24.8)	28.6 (25.7–31.5)
Women	35.7 (29.5–41.9)	36.5 (29.5–43.5)	36.0 (24.9–47.1)	24.2 (13.6–34.9)	34.6 (30.6–38.5)	
**India rural**						
Men	36.7 (28.6–44.8)	38.3 (30.5–46.1)	39.8 (29.3–50.2)	42.5 (30.9–54.1)	36.7 (32.2–41.2)	38.6 (35.3–41.9)
Women	36.5 (29.6–43.3)	46.9 (39.9–53.9)	46.1 (35.3–56.8)	50.0 (37.8–62.2)	40.2 (35.9–44.4)	
**Nigeria**						
Men	16.9 (10.5–23.3)	23.7 (13.9–33.5)	14.7 (6.1–23.3)	23.2 (14.5–31.8)	18.8 (14.9–22.7)	21.1 (18.8–23.5)
Women	15.6 (11.1–20.1)	26.0 (18.8–33.2)	23.1 (11.2–34.9)	43.3 (32.9–53.8)	22.7 (19.5–26.0)	

**Table 4:** Prevalence of sub-syndromal depression (EURO-D depression not confirmed by ICD1-10).

**Centre**	**Crude prevalence (95% CI)**
Cuba	11.4 (10.3–12.7)
Dominican Republic	13.7 (12.2–15.3)
Puerto Rico	7.8 (6.7–9.1)
Mexico (urban)	15.0 (13.0–17.4)
Mexico (rural)	12.2 (10.3–14.4)
Peru (urban)	14.0 (12.2–15.9)
Peru (rural)	12.5 (10.0–15.5)
China (urban)	2.2 (1.5–3.2)
China (rural)	0.4 (0.2–1.1)
India (urban)	24.8 (22.1–27.6)
India (rural)	25.3 (22.6–28.2)
Nigeria	20.4 (18.1–22.8)
