# Supplementary material for: A comparative cross-cultural study of the prevalence of late life depression in low and middle income countries
Source: J Affect Disord. 2016 Jan 15;190:362–8. doi: 10.1016/j.jad.2015.09.004 (PMC4679114; doi:10.1016/j.jad.2015.09.004)
Supplement: Supplementary file 1 — Supplementary material [file mmc1.docx]

**Supplementary figure 1 – Forest plot of crude prevalence of depression (ICD-10 and EURO-D) across sites with pooled estimates.**
